# Antioxidative Effect of *Amomum testaceum* Ridl. Extract for Protecting against Vascular Dementia

**DOI:** 10.1155/2022/1572527

**Published:** 2022-12-29

**Authors:** Pratchaya Kaewkaen

**Affiliations:** ^1^College of Research Methodology and Cognitive Science, Burapha University, Mueang, Chonburi 20131, Thailand; ^2^Animal Cognitive Neuroscience Laboratory (ACoN), Cognitive Science & Innovation Research Unit (CSIRU), Burapha University, Mueang, Chonburi 20131, Thailand

## Abstract

Vascular dementia is caused by decreased blood flow to the brain, which leads to neuronal damage and subsequent death. Therefore, development of alternative neuroprotective agents is critical. This study is aimed at investigating the effects of *Amomum testaceum* Ridl. extract or Siam cardamom extract (SCE) on oxidative markers in a rat model of vascular dementia. The phenolic content of SCE, represented as gallic acid equivalent (mg/100 GAE), was discovered to be 128.56 ± 0.58 mg/100 GAE. The EC50 values for the 2,2-diphenyl-1-picrylhydrazyl radical scavenging activities of SCE were 32.48 ± 0.39 *u*g/mL. In addition, the reducing power of SCE, via a ferric reducing antioxidant power assay, was also determined, with an EC50 value of 142.55 ± 0.56 *u*g/mL. SCE was administered orally to adult male Wistar rats weighing 250–300 g at doses of 100, 300, and 500 mg/kg over the course of 14 days; then, the rats underwent surgery of the right middle cerebral artery, producing an occlusion imitating vascular dementia in a controlled environment. All rats were euthanized to obtain brain tissue for biochemical testing and analysis. The results showed that malondialdehyde decreased and superoxide dismutase, catalase, and glutathione peroxidase increased at all doses (100, 300, and 500 mg/kg) of SCE (*P* < 0.05). In addition, SCE was shown to lower the expression level of S100B, a marker of neurologic injury (*P* < 0.05). The free radical scavenging and anti-inflammatory capabilities of SCE suggest that it has the potential to be used as a food supplement to protect against oxidative damage in vascular dementia. However, further clinical investigations are essential to elucidate this in clinical trials.

## 1. Introduction

Oxidation is an essential source of energy for all living organisms, including humans. Dementia, stroke, cancer, cardiovascular disease, hypertension, and age-related cognitive decline have all been linked to elevated levels of oxygen-derived free radicals or oxidants [1]. Residual oxygen species, which are constantly produced *in vivo* when oxygen is reduced by single electrons, can cause significant damage to cell components such as lipids, proteins, and DNA [2]. In addition, reactive oxygen species (ROS) have been linked to the pathophysiology of other neurodegenerative diseases, such as Parkinson's disease. Reoxygenation during reperfusion delivers oxygen critical for neuronal survival; however, this oxygen also functions as a substrate for a variety of enzyme oxidation activities in the brain that result in reactive oxidant formation during an ischemic cascade in the brain arteries. Because they provide protection against the harm caused by free radicals, antioxidants are essential components of the cell structure. Therefore, developing an approach for reducing oxidation processes is critical for preventing neuronal cell deterioration.

Ischemia of the brain generates inflammation in response to necrotic cells, which is followed by the creation of ROS; however, much is still unknown about this process. These proinflammatory cytokines drive microglia, the immune cells that are normally found in the brain, to become further activated. This results in an increase in the microglial-dependent production of proinflammatory cytokines, which in turn activates adhesion molecules in the brain [3]. S100 calcium-binding protein B (S100B) is a calcium-binding cytoplasmic protein that is predominantly produced by glia. It is thought to be a possible diagnostic tool for injury to the brain or spinal cord, particularly in the context of an inflammatory response. Patients who are suffering from a wide variety of pathological illnesses and neurodegenerative disorders, including cerebral ischemia or stroke, traumatic brain injury (TBI), and Alzheimer's disease, are included in this group and have been found to possess elevated levels of S100B in their cerebrospinal fluid and peripheral blood. These elevated S100B levels occur because brain trauma causes astrocytes to release S100B into their surrounding environment [4, 5]. This protein may be active in the process of neurites amplifying melanoma cell proliferation, alongside the activation of Ca^2+^ flux, inhibition of PKC-mediated phosphorylation, astrocytosis, axonal proliferation, and inhibition of microtubule assembly in the developing central nervous system. S100B has a neuroprotective effect, and the corresponding S100B and cytokine levels reach their highest within the first 24 h following severe TBI. Subsequently, their levels begin to gradually decline. As a result, S100B might be helpful as an outcome predictor in patients with severe TBI [6].

Polyphenols are bioactive chemicals found in plants. They are regarded significant because of their contribution to human health and prevention of chronic diseases. Polyphenols are found in various plant-based foods. Interaction with food matrices can either hinder or improve the accessibility and availability of nutrients. There are many types of Thai medicinal plants that are used as household herbs. *Amomum testaceum* Ridl., or cardamom, is a perennial plant belonging to the *Zingiberaceae* family and one of the 18 species of the *Amomum* genus. Cardamom is mostly composed of volatile oils that stimulate gastric juice secretion and exert antiemetic effects. According to numerous studies, cardamom oil primarily contains 1,8-cineole (20–60%) and *α*-terpinyl acetate (20–55%) as key constituents [7]. Oils and extracts from spices that are frequently used to flavor food are efficient sources of natural antioxidants; consequently, they are employed as nutraceuticals because of the evident hydroxyl groups found in their phenolic components. Cardamom has been used to treat several illnesses, such as asthma, indigestion, and congestive jaundice. Numerous pharmacological properties of cardamom have been discovered, including antioxidant, anti-inflammatory, anticancer, and antibacterial activities [8]. Therefore, the purpose of this study was to determine the effect of Siam cardamom extract (SCE) in a rat model of vascular dementia initiated by middle cerebral artery occlusion. Additionally, I investigated the underlying mechanisms of the corresponding oxidative markers and anti-inflammatory effects of SCE on S100B.

## 2. Materials and Methods

### 2.1. Plant Preparation

Cardamom fruits were collected in Chanthaburi Province, Thailand. The voucher specimen was deposited in the herbarium of the Animal Cognitive Neuroscience Laboratory (ACoN) at Burapha University. These fruits were dehydrated and ground into a powder. An altered form of the sonication procedure was to extract the materials using 80% ethanol as the solvent. The extraction was performed twice on a total of 2 g of powdered materials using 10 mL of solvent. It was then sonicated in the dark for half an hour. The homogenate sample was then centrifuged for 15 minutes at 4 degrees Celsius and 10,000 times its original mass to collect the supernatant. Finally, the supernatant was concentrated to 0.2 g/mL via a rotary vacuum evaporator. The concentrated supernatant (0.2 g/mL) was employed as the extract. This was done so that total phenolic components and antioxidant capability could be evaluated.

### 2.2. Determination of Total Phenolic Compound

The total phenolic compound concentration was measured by a modified Folin-Ciocalteu colorimetric method [9]. Briefly, 1.58 mL of distilled water was added to a test tube holding 1 milligram of diluted SCE. The 100 *μ*L of Folin-Ciocalteu reagent was added, and the tube was mixed and allowed to stand for five minutes at room temperature. The absorbance at 760 nm was measured after 120 minutes at room temperature after adding 2 ml of 7.5% (w/v) Na_2_CO_3_. Comparing the absorbance of the sample to the standard curve of the gallic acid solution yielded the total phenol concentration. The gallic acid equivalent is reported in milligrams per gram of extract [10].

### 2.3. Determination of Antioxidant Activity by 2,2-Diphenyl-1-Picrylhydrazyl (DPPH) Method

The scavenging activity of DPPH free radicals was determined according to a previously reported method [11]. Briefly, 50 *μ*L of the sample and 100 *μ*L of 0.2 mM DPPH solution were mixed for 1 min, and then left in the dark at 22°C temperature for 30 min. The corresponding absorbance was measured at a wavelength of 517 nm using a microplate reader. Ascorbic acid was used as the positive control. The percentage of DPPH removed from the control reaction was calculated using equation as follows:

(1) $$\begin{array}{c} \text{The antiradical activity }\left(\text{AA}\right)\%=100-{\left(\left(\text{Abs}:\text{sample}-\text{Abs}:\text{empty sample}\right)/\text{Abs}:\text{control}\right)}^{*}100. \end{array}$$

### 2.4. Determination of Antioxidant Activity by Ferric Reducing Antioxidant Power (FRAP)

Modifications were made to Benzie's method [12]. The prior description of the ferric reducing antioxidant power assay used to determine total antioxidant capacity was based on the reduction of Fe^3+^. The stock solutions contained 300 mM acetate buffer (3.1 g C_2_H_3_NaO_2_.3H_2_O and 16 mL C_2_H_4_O_2_), pH 3.6; a 10 mM tripyridyltriazine (TPTZ) solution in 40 mM HCl; a 20 mM FeCl_3_.6H_2_O solution. Mixing 25 mL acetate buffer, 2.5 mL TPTZ solution, and 2.5 mL FeCl_3_.6H_2_O solution yielded a fresh working solution. The plant extract (10 *μ*L) in 1 mL of distilled water was allowed to react for 10 minutes at 37 degrees Celsius with 1.8 mL of the FRAP solution. The absorbance of the examined solution was measured at 593 nm. The results were given in M of ascorbic acid per 100 g of fresh weight.

### 2.5. Experimental Protocol

The Nomura Siam International Co., Ltd. in Bangkok, Thailand was provided with 8-week old adult male Wistar rats weighing 250–300 g. All animals were housed in polycarbonate cages (four rats per cage) with a 12 : 12 h light/dark cycle. There was no restriction on the amount of food or water that the animals could consume.

### 2.6. The Experimental Study Design

All animals were randomly assigned to one of seven groups (*n* = 8 animals per group) as follows:

*Group I*. Control group that received no treatment.

*Group II*. Sham group: placebo surgery group.

*Group II*. Vehicle treated group: all rats in this group had a standard diet and were treated with vehicle (distilled water).

*Groups III-IV*. Positive control: rats were treated with piracetam (200 mg/kg BW) and donepezil (3 mg/kg BW), respectively.

*Groups V-VII*. SCE-treated groups: rats were treated at doses of 100, 300, and 500 mg/kg BW, respectively.

**Figure 1:**
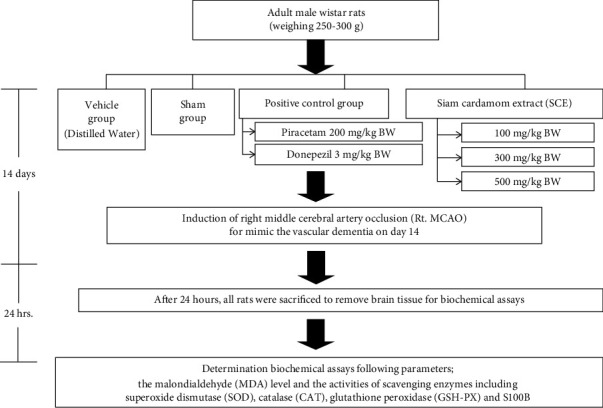
Schematic of the interventions and evaluations performed here.

All rats were treated with the assigned substance once daily by oral gavage for 14 days. All rats were sacrificed 24 hours after the last treatment to remove the brain as described in Figure 1.

### 2.7. Induction of Middle Cerebral Artery Occlusion

The operation for occluding the right middle cerebral artery was done using a modified Longa technique [13]. Anesthesia was administered to rats at 50 mg kg/BW thiopental sodium. Both the external carotid artery and the common carotid artery on the right side were revealed and ligated proximally via a ventral midline neck incision. The anterior cerebral artery, the middle cerebral artery, and the posterior cerebral artery were also ligated. The USS DG^™^ silicone-coated nylon monofilament (4-0) suture was heated to induce a round tip, and then inserted through an arteriectomy in the carotid artery just below the bifurcation of the carotid artery. It was then moved about 17 millimeters into the interior carotid artery distal until mild resistance was encountered. The beginning of the anterior cerebral artery, the middle cerebral artery, and the posterior connecting artery were all thus obstructed. After the suturing was complete, the rats were put back in their cages where they had access to food and water. A povidone-iodine solution diluted to 10% was injected into the incision sites. After 24 hours, all rats were sacrificed so that brain tissue could be removed for biochemical assays.

### 2.8. Biochemical Assays

*Preparation of Tissue Homogenates.* All animals were anesthetized with an intraperitoneal injection of 50 mg/kg BW pentobarbital sodium 24 hours after inducting the surgery. Isolated brains were kept cold in ice buckets. The tissues were then homogenized in four liters of 1.15% KCl using a glass Potter-Elvehjem homogenizer.

### 2.9. Determination of Lipid Peroxidation

The amount of malondialdehyde (MDA), a lipid peroxidation product, was measured using the thiobarbituric acid reaction. To summarize, 0.1 mL of processed tissue samples were mixed with 1.5 mL of acetic acid (20%), 1.5 mL of thiobarbituric acid (0.8%), and 0.2 mL of sodium dodecyl sulphate (8.1%) before being heated for 60 minutes at 100 degrees Celsius. After the combination was cooled by passing it through running tap water, it was given a vigorous vortex in which 5 mL of n-butanol-pyridine (15 : 1) and 1 mL of distilled water were mixed together. After 10 minutes of centrifugation at 4000 rpm, the organic layer was separated, and a spectrophotometer was used to measure the absorbance at 532 nm. The amount of MDA that was contained in the tissue was expressed as nmol per gram. The colorimetric approach developed by Tang et al. was used to determine the protein content [14].

### 2.10. Scavenging Enzymes Assay

#### 2.10.1. Assay of Catalase (CAT) Activity

The CAT concentration was measured using the method of Aebi et al. [15]. One minute was used for the enzyme samples or standard enzyme solution to react with hydrogen peroxide. The reaction was then terminated using a sulfuric acid solution. After adding the combination to a potassium permanganate solution, excess peroxide that had not been digested by catalase was allowed to react with it. Upon addition of permanganate, the reaction with peroxide was quantified photometrically at 515 nm. Absorbance at 515 nm was plotted against the catalase activity to produce a standard curve. Catalase units per mg protein were used to represent the findings.

#### 2.10.2. Assay of Superoxide Dismutase (SOD) Activity

SOD activity was determined at 550 nm based on the corresponding superoxide radical-dependent suppression of the cytochrome C-reduction rate. The final concentrations in a 1 mL system mixture were as follows: 50 mM potassium phosphate, 0.1 mM ethylenediaminetetraacetic acid (EDTA), 0.01 mM cytochrome C, 0.05 mM xanthine, 0.005 unit xanthine oxidase, and 1 unit SOD solution or sample. SOD solution was used as a control for enzyme activity. The standard curve is presented as the percentage inhibition of SOD activity. Unit activity was defined as the quantity of enzyme required to reduce cytochrome C by 50% in a linked system employing xanthine-xanthine oxidase at pH 7.8 and 25°C. The results are expressed as SOD activity units per mg protein [16].

#### 2.10.3. Assay of Glutathione Peroxidase (GSH-Px) Activity

The activity of GSH-Px was assessed using Wendel's technique, in which activity was evaluated indirectly through a coupled reaction with GSH reductase. In this assay, oxidized GSH is recycled to its reduced form by GSH reductase and NADPH after the reduction of hydrogen peroxide by GSH-Px. NADPH oxidation to NADP+ resulted in a reduction in absorbance at 340 nm. The standard curve is displayed as the ratio of GSH-Px activity to the rate of absorbance 340 nm per minute. The final 1 mL of the reaction mixture contained the standard enzyme GSH-Px solution or 17 *μ*L of a homogenized brain sample together with 48 mM sodium phosphate, 0.38 mM EDTA, 0.12 mM NADPH, 0.95 mM sodium azide, 3.2 units of GSH reductase, 1 mM GSH, and 0.02 mM DL-dithiothreitol. A standard curve versus GSH-Px activity was plotted. The quantity of enzyme needed to catalyze the oxidation of 1 mol of GSH to GSSG per minute at pH 7, and 25°C is known as a unit activity. The data are presented as grams of protein per GSH-Px [17, 18].

### 2.11. Assessment of Brain Inflammation Biomarkers via S100B

S100B isolated from bovine brain and purchased from Sigma was diluted in phosphate buffer solution to a concentration of 1 mg/mL, filtered, and stored in aliquots at a temperature of 80°C. This dilution corresponded to a dimer concentration of 50 *μ*M S100B. To carry out NF-*κ*B loss-of-function investigations, neuronal cells cultured in the neurobasal-B27 medium were supplemented with S100B; as an alternative, neuronal tissue was treated with S100B with 2.5 *μ*M sulfasalazine for 24 h. To carry out receptor for advanced glycation end products (RAGE) blocking tests, neuronal cultures were incubated with an anti-RAGE antibody (MAB 5328; Chemicon), which was designed to precisely target the RAGE extracellular domain. Neuronal tissues were treated with S100B after being exposed to either 1.5 kg of anti-RAGE or unrelated mouse IgG for a period of 2 h [19].

### 2.12. Statistical Analysis

The data are presented as means with standard deviation of the mean (SEM). One-way analysis of variance (ANOVA) and the Student Newman-Keuls test was used for statistical comparisons across groups. All analyses used SigmaStat version 4.0. The level of statistical significance was *P* < 0.05.

### 2.13. Ethics Statements

This research was performed while reducing the amount of pain caused to the animals in accordance with standard guidelines recognized by the European Commission. The techniques for conducting the experiment were approved by the Institutional Animal Care and Use Committee (IACUC) of Burapha University (Approval Number 9/2564).

## 3. Results

### 3.1. Total Phenolic Compound, DPPH Scavenging Capacity, and FRAP Activity of SCE

The phenolic content is expressed as gallic acid equivalent (mg/100 GAE) in SCE and was found to be 128.56 ± 0.58 mg/100 GAE. The DPPH and FRAP assays were used to evaluate the antioxidant impact.  Table 1 presents the findings of the study. The EC_50_ value for the DPPH radical scavenging activities of SCE is 32.48 ± 0.39 *u*g/mL. In addition, the reducing power of SCE via a FRAP assay was determined with an EC_50_ of 142.55 ± 0.56 *u*g/ml.

**Figure 2:**
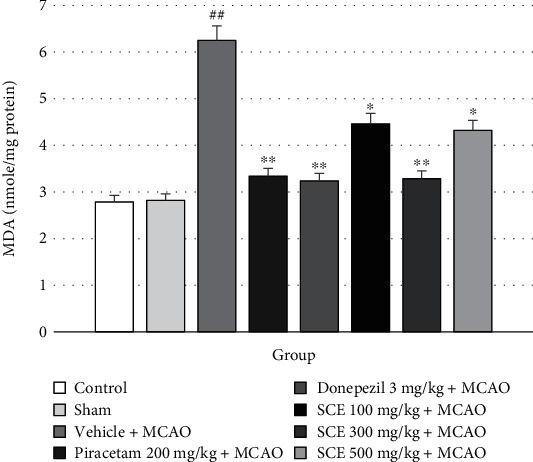
Effect of Siam cardamom extract on the level of malondialdehyde (MDA) in the cerebral cortex. Data are presented as mean ± SEM (*n* = 8/group). ^∗^*P* value < 0.05 compared with vehicle plus MCAO, ^∗∗^*P* value < 0.01 versus vehicle plus MCAO, and ^##^*P* value < 0.01 versus control group.

The effect of SCE on oxidative stress markers and inflammatory markers was also investigated. The malondialdehyde level is commonly known to be a marker of oxidative stress. The results showed that MDA was significantly decreased in the SCE-treated group (100, 300, and 500 mg/kg) versus the vehicle+MCAO group (^∗^*P* < 0.05 and ^∗∗^*P* < 0.01, respectively). The results are shown in Figure 2.

**Figure 3:**
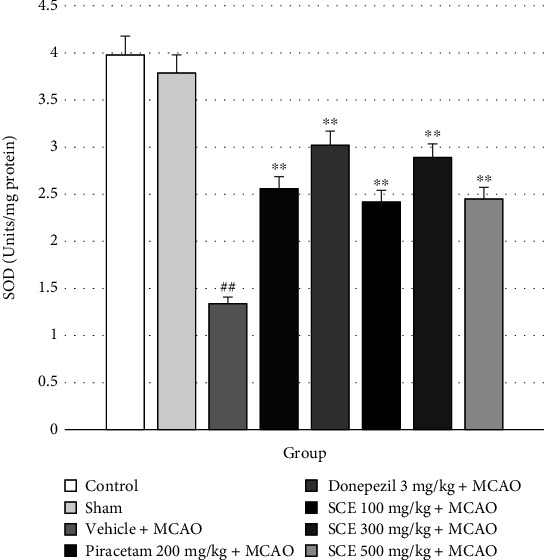
Effect of Siam cardamom extract on the level of superoxide dismutase (SOD) in cerebral cortex. Data are presented as the mean ± SEM (*n* = 8/group). ^∗∗^*P* value < 0.01 versus vehicle plus MCAO; ^##^*P* value < 0.01 versus control group.

Superoxide dismutase (SOD) acts as an antioxidant. The transformation of the superoxide anion into hydrogen peroxide is catalyzed by SOD. SOD is then transformed into oxygen and water by catalase or GSH peroxidase.  Figure 3 shows the effect of SCE on oxidative stress markers of SOD activities. Interestingly, elevated levels of SOD activity in rat brain were found in rats administered SCE at 100, 300, and 500 mg/kg.

**Figure 4:**
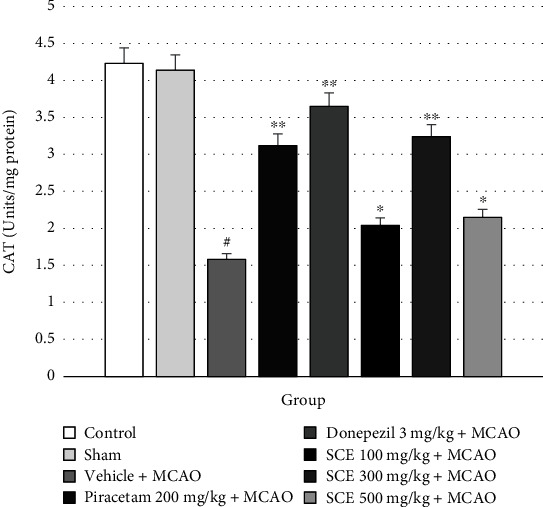
Effect of Siam cardamom extract on the level of catalase (CAT) in the cerebral cortex. Data are presented as mean ± SEM*n* = 8/group. ^∗^*P* value < 0.05 versus vehicle plus MCAO, ^∗∗^*P* value < 0.01 versus vehicle plus MCAO, and ^#^*P* value < 0.05 versus control group.

**Figure 5:**
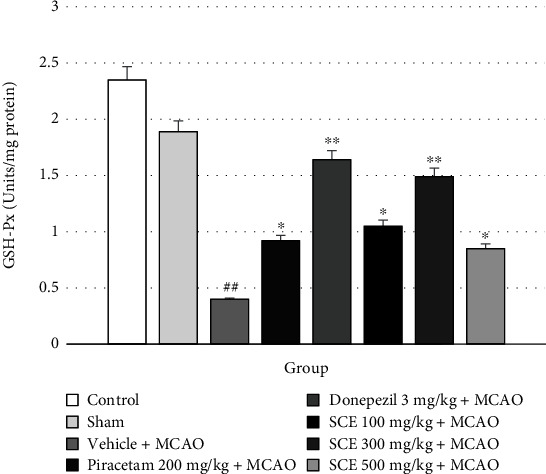
Effect of Siam cardamom extract on the activity of glutathione peroxidase (GSH-Px) in the cerebral cortex. Data are presented as mean ± SEM*n* = 8/group. ^∗^*P* value < 0.05 versus vehicle plus MCAO, ^∗∗^*P* value < 0.01 versus vehicle plus MCAO, and ^##^*P* value < 0.01 versus control group.

The alterations of oxidative stress markers CAT and GSH-Px in rat cerebral cortex are shown in Figures 4 and 5, respectively. These data indicate that cerebral ischemic rats exposed to piracetam and donepezil had enhanced activities of CAT and GSH-Px in the cerebral cortex.

### 3.2. Effect of Siam Cardamom Extract on S100B

**Figure 6:**
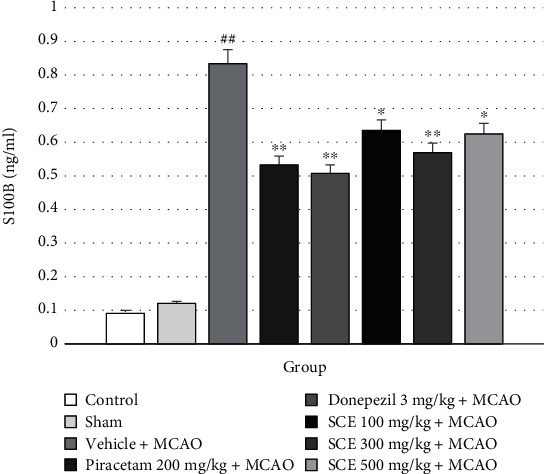
Effect of Siam cardamom extract on the level of S100 calcium-binding protein (S100B). Data are presented as mean ± SEM*n* = 8/group. ^∗^*P* value < 0.05 versus vehicle plus MCAO, ^∗∗^*P* value < 0.01 versus vehicle plus MCAO, and ^##^*P* value < 0.01 versus control group.

Elevated levels of S100B have also been detected in brain regions associated with cerebral injury. S100B is glial-specific and is expressed primarily by astrocytes. Here, rats were treated with SCE at doses of 100, 300, and 500 mg/kg and had decreased S100B levels (*P* value < 0.05 compared with vehicle plus MCAO); see Figure 6.

## 4. Discussion

As the number of elderly people in the global population continues to rise, the incidence of vascular dementia, which is brought on by cerebral ischemia, is continuously rising each year. Consequently, this can result in significant public health challenges in the future. Vascular dementia is characterized by a gradual decline in memory and cognitive abilities. This condition can be caused by a disruption in blood flow to the brain or by a breakdown in the system of blood vessels that supply blood to the brain. Therefore, the use of synthetic pharmaceuticals as a potentially effective alternative therapeutic for preventing or curing dementia has been an attractive emerging area for research. It is a well-established fact that polyphenolic compounds, and flavonoids in particular, can have a generally positive impact in vivo and, specifically, increase antioxidant activity in the body. SCE has the potential to prevent diseases because it includes several antioxidants that contribute to the defensive mechanisms against such diseases. Recent studies have demonstrated that (poly) phenolic compounds are the richest essential phytochemicals found in plants. These phytochemicals can have a functional purpose and improve overall health; consequently, a dietary deficiency of these phytochemicals may lead to an increased susceptibility to several illnesses. Some phytonutrients are powerful antioxidants that can influence the activation of metabolic pathways and help detoxify carcinogens [20]. Further, phytonutrients have been linked to a reduced risk of cancer development. Polyphenols are complex bioactive molecules found in high quantities in the diets of both humans and animals, particularly in plant-based foods. A number of studies have established that polyphenols possess health-promoting characteristics. These properties have been demonstrated because of their ability to scavenge free radicals and their wide range of bioactivity.

The current study suggests that SCE has antioxidant effects in a rat model of vascular dementia. This was demonstrated by a decrease in the MDA level and an increase in the expression of S100B, in addition to an increase in the activities of SOD, CAT, and GSH-Px in the cerebral cortex of the MCAO rats. The middle cerebral artery was blocked in the animal model used in this study to simulate the effects of cerebrovascular disease. In particular, this model simulated the effects of acute stroke with the understanding that this artery is the predominantly affected artery in acute stroke. MCAO occurs in four major directions, designated as M1, M2, M3, and M4, which originate from the internal carotid artery. These arteries deliver blood to deeper areas of the brain, including the thalamus, caudate nucleus, and internal capsule [21]. The frontal, temporal, and parietal regions of the brain receive blood from these arteries. It has been suggested that oxidative stress is the likely mechanism underlying MCAO-induced neurotoxicity. In general, oxidative stress can be understood as an imbalance in the production of ROS and failure of the natural antioxidant system in the body. This is the fundamental definition of oxidative stress. The pathological state known as oxidative stress is brought on by either an insufficient supply of antioxidants or an excessive production of ROS. Under these circumstances, the organ of the body most susceptible to damage is the brain. The brain has the highest oxygen requirement of any organ in the body, and it also contains redox-active metals, such as iron and copper, which catalyze the creation of ROS. Additionally, the high concentrations of polyunsaturated fatty acids found in the brain make it an ideal substrate for lipid peroxidation [22].

SOD converts superoxide anions to H_2_O_2_, which is then converted to a toxic hydroxyl radical via the Haber-Weiss reaction, GSH-Px, or CAT. It has been suggested that accumulation of H_2_O_2_ has neurotoxic effects. These findings suggest that the antioxidant and anti-inflammatory effects of SCE may be due to its antioxidant activity, which stimulates the activity of scavenging enzymes that protect neurons from oxidative damage. The impact of the aqueous extract of large cardamom in preventing vasculature remodeling and oxidative stress in N-nitro-L-arginine methyl ester- (L-NAME-) induced hypertension may be attributed to antioxidant activity and nitric oxide level restoration [23]. Additionally, it has been reported that cardamom protects the myocardium and exerts cardioprotective effects through free radical scavenging and antioxidant activity in Wistar male albino rats treated with cardamom extracts (100 and 200 mg/kg orally) [24].

**Figure 7:**
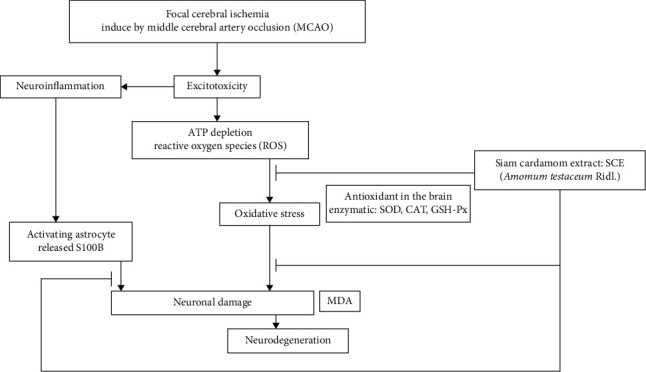
Potential mechanism responsible for the ameliorating impact of Siam cardamom extract (*Amomum testaceum* Ridl.) on oxidative damage and decreased S100B levels during vascular dementia protection. As a calcium-sensitive protein, S100B controls intracellular processes such as signal transduction and inhibits the survival of damaged neurons.

S100B is involved in various physiological processes, operates as a modulator of intracellular activities, and performs a regulatory extracellular role. This includes signal transmission, which impedes cell survival, proliferation, and differentiation. It has been reported that in astroglia, this Ca^2+^-binding protein is overexpressed to a significant degree. S100B has been discovered in a wide variety of neurodegenerative diseases and appears to play a role in the progression of the pathophysiological processes of Alzheimer's disease during the early stages of this disease [25, 26]. According to earlier studies, the most common consequences of cerebral ischemia are brain cell death and fluid accumulation in the brain. S100B can be used as a biomarker for damage to the central nervous system because it is found in higher amounts in patients who have suffered damage to their brain and nerves. In this study, we demonstrated that SCE extract reduced the amount of S100B in the cerebral cortex (compared to vehicle+MCAO, *P* value < 0.05). The potential mechanism responsible for the ameliorating impact of SCE on oxidative damage and decreased S100B levels during vascular dementia protection as shown in Figure 7.

In this study, we used positive control groups that were treated with piracetam or donepezil. Piracetam has been shown to be a helpful treatment for cognitive loss associated with aging and dementia. Disruption of brain function often manifests itself in a much more active state. Piracetam is a powerful antioxidant, a cerebral neuroprotector, an enhancer of neuronal metabolic activity, and an agent that promotes integration into the brain [27]. Consequently, it is associated with age-related neurochemical deficiencies in the brain that lead to cognitive dysfunction. These deficits are associated with cognitive dysfunction. Further, piracetam alters membrane characteristics by interfacing with the polar head moieties of the phospholipid bilayer. This is of relevance due to the fact that many of these neurochemical deficiencies are dependent on changes in membrane characteristics, such as fluidity [28]. Possible treatment of these deficiencies affects the membrane characteristics by engaging with the polar head moieties of the phospholipid bilayer. Donepezil is a second-generation cholinesterase suppressor, and its therapeutic effects are demonstrated by the reversible suppression of acetylcholinesterase [29]. In this study, a positive response in both positive control groups was demonstrated in this animal model of vascular dementia. Finally, SCE did not appear to have a dose-dependent impact in this particular investigation. It is highly likely that the recorded parameters were obscured by the impact of other drugs, as well as by the nonlinear relationship between these characteristics and SCE concentrations. Both factors contributed to the results obtained.

## 5. Conclusion

Vascular dementia imposes a substantial financial cost, and the current therapeutic approach is inadequate; therefore, its prevention is crucial. SCE extract can exert protective effects against oxidative stress. This was demonstrated by the reduced MDA levels, as well as the increased CAT and GSH-Px levels. Additionally, SCE caused a reduction in S100B levels in brain regions associated with vascular dementia. Therefore, SCE could be researched further as a strong candidate for development of a therapeutic method for the treatment of vascular dementia.

## Figures and Tables

**Table 1:** Total phenolic compound, scavenging capacity of DPPH, and FRAP activity of SCE.

No.	Substance	Total phenolic compounds (mg/100 GAE)	^∗^EC_50_ of DPPH (*u*g/mL)	^∗^EC_50_ of FRAP (*u*g/mL)
1	SCE	128.56 ± 0.58	32.48 ± 0.39	142.55 ± 0.56
2	Vitamin C	—	96.36 ± 0.42	102.74 ± 0.94

Remark: these values represent the mean ± standard deviation of three independent investigations. Total phenolic content is given as mg gallic acid equivalent/g. ^∗^: the EC50 value is the effective concentration that elicits a reaction halfway between baseline and maximum after a defined exposure time.

## Data Availability

The data that support the findings of this study are available from the corresponding author, upon reasonable request.
